# Association of body mass index with pathologic agreement of preoperative and postoperative tumor grade in endometrial cancer

**DOI:** 10.1007/s00404-024-07829-z

**Published:** 2024-11-15

**Authors:** Samantha Taylor, Peter Scalia, Raanan Meyer, Melica Nourmoussavi Brodeur, Shannon Salvador, Susie Lau, Walter Gotlieb, Gabriel Levin

**Affiliations:** 1 Department of Obstetrics and Gynecology, McGill University, Montreal, QC Canada; 2 School of Medicine, McGill University, Montreal, QC Canada; 3 Division of Minimally Invasive Gynecologic Surgery, Department of Obstetrics and Gynecology, Cedars Sinai Medical Center, Los Angeles, CA USA; 4 Division of Gynecologic Oncology, Jewish General Hospital, McGill University, Montreal, QC Canada; 5 The Department of Gynecologic Oncology, Hadassah Medical Center, Faculty of Medicine, Hebrew University of Jerusalem, Jerusalem, Israel

**Keywords:** Body mass index, Endometrial biopsy, Endometrial carcinoma, Grade, Obesity

## Abstract

**Objective:**

We aim to study association of BMI of EC patients, with the level of agreement between preoperative and postoperative tumor grade.

**Methods:**

A retrospective study. We included patients with EC diagnosed in an outpatient clinic which had surgical staging as in our division. We categorized patients into BMI categories according to the World Health Organization; (BMI < 18.5 kg/m^2^), (BMI 18.5–24.9 kg/m^2^), (BMI 25–29.9 kg/m^2^), (BMI 30–34.9 kg/m^2^), (BMI 35–39.9 kg/m^2^), and (BMI ≥ 40 kg/m^2^). We further dichotomized the study population for obesity, defined as BMI ≥ 30.0. We analyzed agreement between preoperative and postoperative tumor grade, stratified by patient’s BMI.

**Results:**

Overall, 623 women met study inclusion criteria, with a median age of 64 [interquartile range (IQR) 57–72]. Among the study cohort, the median BMI was 30.7 [IQR 25.6–38.8], with 330 (53.0%) patients being obese. EC grade 1 was diagnosed preoperatively in 353 (56.7%), grade 3 in 148 (23.8%), and grade 2 in 122 (19.6%). Endometrioid histology was diagnosed in 463 (74.3%), serous in 78 (12.5%), mixed histotype in 51 (8.2%), clear cell in 20 (3.2%) and carcinosarcoma in 11 (1.8%). In 68.7% (*n* = 428), there was no change in postoperative grade, and in 24.9% (*n* = 155), there was upgrading of tumor, and in 6.4% (*n* = 40), there was a tumor downgrade. There were 3 (0.5%) cases in which no tumor was found on final pathology. The rate of no change was higher in preoperative grade 3 (89.9%) vs. grades 1 (63.5%) and grade 2 (58.2%), *p* < .001). There was no difference in grading agreement when obese patients were compared to non-obese, *p* = .248. There was no difference in grading agreement when comparing the various BMI categories, with no change proportion ranging between 58.2% in BMI 30.0–34.9 mg/kg^2^ and 79.7% in BMI 35.0–39.9 mg/kg^2^, *p* = .104. ROC analysis of BMI as predictor of no-change yielded an area under the curve of 0.466 (95% confidence interval 0.418–0.515) with a maximal performance at a BMI of 33.8 mg/kg^2^. The agreement between preoperative and postoperative tumor grade among all patients was kappa = 0.517. The agreement did not differ when compared between obese patients (kappa = 0.456) and non-obese (kappa = 0.575).

**Conclusion:**

Our study found no significant association between BMI and the agreement between preoperative and postoperative tumor grading in EC.

## What does this study add to the clinical work

**Table:** 

There is no association between BMI and the agreement between preoperative and postoperative tumor grading in endometrial carcinoma.	

## Introduction

Endometrial cancer (EC) is the most common gynecologic malignancy in the United States, with approximately 67,880 new cases projected to be diagnosed in 2024 with approximately 13,250 deaths [1]. EC poses significant health burden as it has the fastest increasing mortality and is the only cancer for which survival has decreased over the past decades [1–3].

Obesity is known to impact the outcomes of gynecological malignancies [4]. Moreover, obesity is a well-established risk factor for the most common histotype of EC, primarily due to the excessive production of unopposed estrogen from abundant adipose tissue [5, 6]. Recent epidemiological data indicates that in the United States, nearly 1:2 adults will be obese by 2030 and 1:4 adults will have severe obesity [7].

Obesity is not solely a risk factor for EC, as has implications for EC screening and management. While ultrasound measurement of the endometrial thickness is pivotal in assessment of abnormal vaginal bleeding in postmenopausal women, it was shown that obesity decreased the accuracy of endometrial stripe thickness measurement [8].

Moreover, it is not established whether obesity has any impact on the accuracy of endometrial biopsy in prediction of postoperative tumor grade on final pathology [9]. As EC tumor grade on preoperative endometrial biopsy as implications on patient preoperative workup, as performance of preoperative imaging studies, it is of utmost importance to clarify the association of body mass index (BMI) with the accuracy of preoperative endometrial biopsy in predicting postoperative final tumor grade.

We aim to study association of BMI of EC patients, with the level of agreement between preoperative and postoperative tumor grade.

## Materials and methods

### Study population

This is a retrospective study, conducted at the division of gynecologic oncology at a tertiary care hospital. We included patients with EC diagnosed in an outpatient clinic which had surgical staging as in our division. We included all histotype of EC. We did not include patients with a preoperative diagnosis of endometrial intraepithelial neoplasia. We excluded patients without a record BMI at time of preoperative biopsy. We categorized patients into BMI categories according to the World Health Organization; Underweight (BMI < 18.5 kg/m^2^), Normal BMI (BMI 18.5–24.9 kg/m^2^), Overweight (BMI 25–29.9 kg/m^2^), Obesity class 1 (BMI 30–34.9 kg/m^2^), Obesity class 2 (BMI 35–39.9 kg/m^2^), and Obesity class 3 (BMI ≥ 40 kg/m^2^). We further dichotomized the study population for obesity defined as BMI ≥ 30.0.

### Study groups and outcomes

A comparison of preoperative vs. postoperative tumor grade was performed. We divided the study cohort into three groups, in accordance with agreement of preoperative vs. postoperative tumor grade: no change, upgrading, and downgrading. The primary outcome measures were the association of BMI with agreement categories.

### Data collection

Medical records were reviewed to obtain age at diagnosis, height, weight and BMI, preoperative endometrial sampling method (endometrial biopsy, dilation and curettage, and others, such as hysteroscopy, cervical biopsy, and endocervical curettage), histology (endometrioid, serous, clear cell, carcinosarcoma, and others), tumor grade (grades 1, 2, and 3), both preoperative and postoperative. Patients were excluded from analysis if they had incomplete medical records for the above-mentioned data. Pathologists specializing in gynecologic oncology pathology reviewed all final surgical pathology specimens and the histologic grade was determined by the International Federation of Gynecology and Obstetrics criteria.

### Statistical analysis

Study groups were compared through univariable analysis. For categorical variables, we used chi-square test, and for continuous variables, we used Mann–Whitney U test. Bonferroni method was executed to identify intergroup significance when more than two groups were compared. Categorical variables were reported as proportions and continuous variables as median [interquartile range]. Received operating curves (ROC) analysis was performed in order to identify the best BMI cut-off for predicting no change in postoperative tumor grading using the Kolmogorov–Smirnov (K–S) metrics.

Cohen’s kappa coefficients were used to compare preoperative and postoperative tumor grades in the various BMI categories and in obese vs. no obese.

Sample size calculation for this retrospective study was as follows: by a previous study, the rate of grade change between endometrial biopsy and hysterectomy was seen in 29.4% [10]. Therefore, 598 patients are required to have a 90% power of detecting, as significant at the 5% level, an increase in the primary outcome measure from 29.4% in the control group to 42% (approximately 40% change in outcome) in the experimental group. All statistical analyses were based on two-tailed hypotheses, and a *p* < 0.05 was considered statistically significant. IBM SPSS Statistics 29.0 was used for statistical analyses.

### Ethical approval

The study was performed in accordance with the Declaration of Helsinki and was approved by the institutional review board.

## Results

Overall, 623 women met study inclusion criteria, with a median age of 64 [interquartile range (IQR) 57–72] (Table 1). Among the study cohort, the median BMI was 30.7 [IQR 25.6–38.8], with 330 (53.0%) patients being obese. The various BMI categories are presented in Table 2. Preoperative diagnostic procedures were as follows: endometrial sampling in 507 (81.4%), dilation and curettage in 75 (12.0%), and 41 (6.6%) with other procedures (Table 1). EC grade 1 was diagnosed preoperatively in 353 (56.7%), grade 3 in 148 (23.8%), and grade 2 in 122 (19.6%). Endometrioid histology was diagnosed in 463 (74.3%), serous in 78 (12.5%), mixed histotype in 51 (8.2%), clear cell in 20 (3.2%) and carcinosarcoma in 11 (1.8%). The postoperative characteristics are presented in Table 3. In 68.7% (*n* = 428), there was no change, in 24.9% (*n* = 155), there was upgrading of tumor, and in 6.4% (*n* = 40), there was a tumor downgrade. There were 3 (0.5%) cases in which no tumor was found on final pathology.

**Table 1:** Patient characteristics

Characteristics	*n* = 623 (%)
*Demographics*	
Age, years	64 [57–72]
Gravidity	2 [1–3]
Parity	2 [1–3]
ASA III/IV	199 (32.0%)
Hypertension	295 (47.4%)
Diabetes	120 (19.3%)
Chronic respiratory disease	66 (10.6%)
*Preoperative parameters*	
Diagnostic procedure	
Endometrial sampling	507 (81.4%)
Dilation and curettage	75 (12.0)
Other	41 (6.6%)
Preoperative grade	
1	353 (56.7%)
2	122 (19.6%)
3	148 (23.8%)
Preoperative histology	
Endometrioid	463 (74.3%)
Serous	78 (12.5%)
Clear cell	20 (3.2%)
Carcinosarcoma	11 (1.8%)
Mixed	51 (8.2%)

Data are *n* (%) or Median (interquartile range)

*ASA* American Society of Anesthesiologists

**Table 2:** Body mass index parameters

Body mass index parameters	*n* = 623 (%)
Body mass index, kg/m^2^	30.7 [25.6–38.8]
> Body mass index, kg/m^2^	
< 18.5	6 (1.0%)
18.5–24.9	128 (20.5%)
25.0–29.9	159 (25.5%)
30.0–34.9	110 (17.7%)
35.0–39.9	79 (12.7%)
> 40	141 (22.6%)
Obese	330 (53.0%)

**Table 3:** Post-operative characteristics

Characteristics	*n* (%)
Postoperative grade	
No cancer	3 (0.5%)
1	250 (40.1%)
2	196 (31.5%)
3	174 (27.9%)
Change in grade following surgery	
No change	428 (68.7%)
Upgrading	155 (24.9%)
Downgrading	40 (6.4%)

**Fig. 1:**
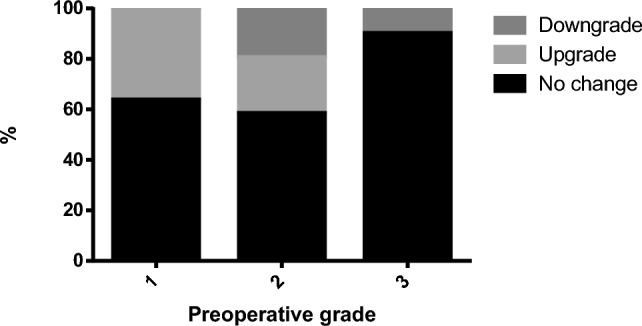
Grading agreement among the various preoperative tumor grades

**Fig. 2:**
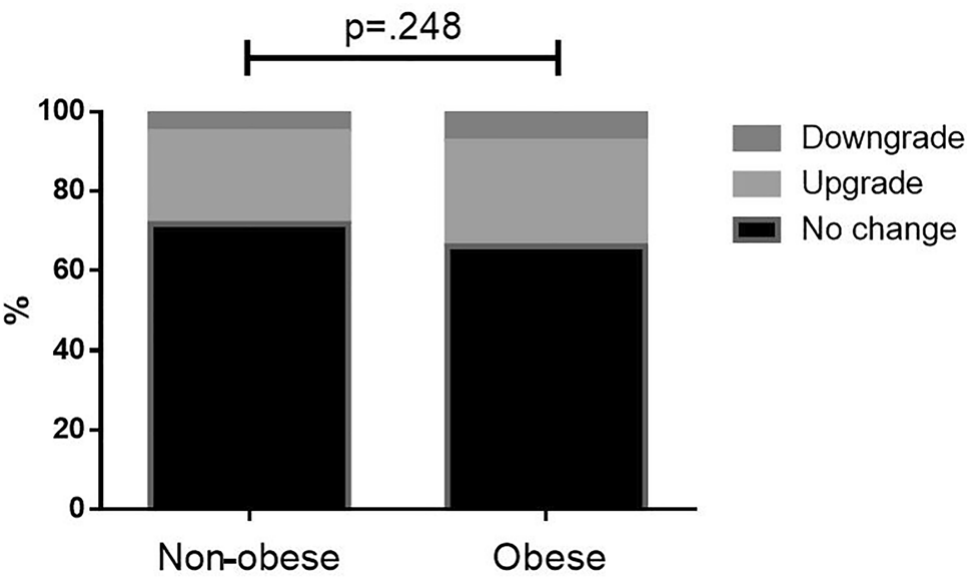
Grading agreement among obese vs. non-obese

**Fig. 3:**
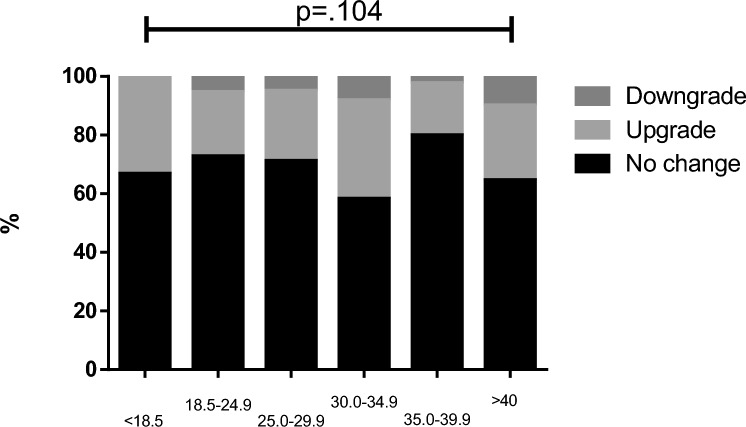
Grading agreement in the various body mass index categories

**Fig. 4:**
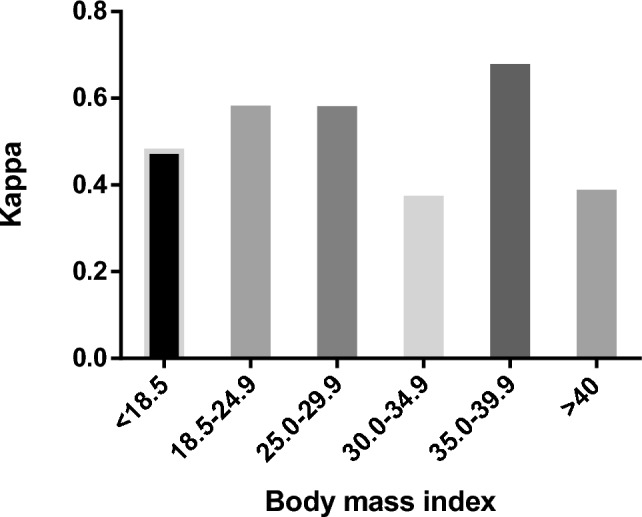
Agreement in the various body mass index categories

Figure 1 presents the proportions of grading agreement in the different preoperative tumor grades. The rate of no change was higher in preoperative grade 3 (89.9%) vs. grade 1 (63.5%) and grade 2 (58.2%), *p* < 0.001). There was no difference in grading agreement when obese patients were compared to non-obese (Fig. 2), with no change in 66.1% of obese patients vs. 71.7% no change in non-obese patients, *p* = 0.248. There was no difference in grading agreement when compared to various BMI categories, with no change proportion ranging between 58.2% in BMI 30.0–34.9 mg/kg^2^ and 79.7% in BMI 35.0–39.9 mg/kg^2^, *p* = 0.104 (Fig. 3). ROC analysis of BMI as predictor of no-change yielded an area under the curve of 0.466 (95% confidence interval 0.418–0.515) with a maximal performance at a BMI of 33.8 mg/kg^2^. The agreement between preoperative and postoperative tumor grade among all patients was kappa = 0.517. It did not differ when compared between obese patients (kappa = 0.456) and non-obese (kappa = 0.575) (Table 4). When stratified by BMI categories, the agreement between preoperative and postoperative varied between kappa = 0.369–0.673 (Fig. 4**)**.

**Table 4:** Kappa coefficient of agreement in the various body mass index categories

Cohort	*n*	Kappa
Overall	623	0.517
Obesity		
Obese	330	0.456
Non-obese	293	0.575
BMI, kg/m^2^		
< 18.5	6	0.478
18.5–24.9	128	0.577
25.0–29.9	159	0.576
30.0–34.9	110	0.369
35.0–39.9	79	0.673
> 40	141	0.383

BMI—Body mass index

## Discussion

In our study, we found that BMI is not associated with agreement between preoperative and postoperative tumor grading in patients with EC. In two out of three patients, the post-operative tumor grade was in an agreement with the preoperative biopsy tumor grade. The rate of grading agreement was higher in preoperative grade 3 when compared to grades 1 and 2.

Accurate tumor grading is crucial for guiding treatment strategies and predicting outcomes in patients with EC. Preoperative tumor grading provides valuable information for initial treatment planning, but its alignment with postoperative findings can significantly impact clinical decision-making.

Obesity has long been established as a risk factor for EC due to its physiological impact on hormone levels, notably through increased unopposed estrogen production from adipose tissue [11–14]. While obesity is associated with heightened EC risk, its role in affecting diagnostic and prognostic precision has been less clear [9], and inconsistencies between preoperative and postoperative grades could potentially alter therapeutic pathways, impacting patient outcomes. Our study underscored the importance of precise grading during the preoperative phase, especially as preoperative grade 3 showed higher agreement rates, suggesting greater reliability in assessing more aggressive forms of EC preoperatively.

Importantly, contrary to the hypothesis that obesity might complicate accurate tumor grading due to factors like tumor biology alteration and diagnostic challenges, our findings indicate no significant association between BMI and the agreement of tumor grades pre- and post-surgery. In most cases, approximately two-third, the tumor grade identified preoperatively remained unchanged postoperatively. However, a notable percentage of cases experienced grade changes, with upgrading occurring in about a quarter of the cases. There was no significant difference in tumor grading agreement between obese and non-obese patients. This suggests that obesity does not significantly affect the consistency of preoperative and postoperative tumor grading.

A previous study also examined the association between BMI and grading agreement between preoperative and postoperative pathology [9]. While that study concluded that obesity does not appear to significantly alter the correlation between preoperative biopsy and final tumor grade, it is important to underline some limitations in that study, as smaller sample size (*n* = 445), and that analysis was performed only in dichotomy between BMI < and > 30 mk/kg^2^. Interestingly, that study found a rather poor agreement level (kappa = 0.2) between pre- and postoperative pathology grading, while in our study, the agreement values were significantly higher.

Furthermore, our analysis demonstrated that BMI is not a strong predictor of agreement in tumor grading, as indicated by the area under the curve (AUC) of 0.466, indicating no predictive power.

Importantly, an Italian study, found that obesity was the only factor in a multivariate analysis lowering the pre- and postoperative tumor grading agreement [15]. However, that study included only 245 patients. Not surprisingly, that study also showed that grade 2 tumor was the tumor grade that most frequently disagreed with the final surgical specimen analysis both in the general and in obese patients.

The impact of BMI on clinical management may go beyond the scope of this study. It was previously suggested that BMI is associated with the risk of having concomitant EC in women with a preoperative biopsy of endometrial intraepithelial neoplasia [16, 17].

Interestingly, our study, along with those previously mentioned [9, 15], reveals a high agreement in grade 3 tumors, suggesting that aggressive tumor phenotypes are more consistently classified preoperatively. This may be due to the notion that grade 3 tumors tend to exhibit less histological variability compared to lower-grade tumors, allowing pathologists to apply clear and uniform criteria for grading.

Of note, the pre-operative endometrial sampling method (blinded biopsy, hysteroscopy guided etc.) may affect the degree of agreement with hysterectomy pathology specimen, with hysteroscopic resection being the most accurate according to studies [18, 19].

Clinically, the implication of our study results, with the limitation elaborated later on, suggests that patient’s BMI should not be a criterion in the considerations of tailoring patient’s management as further pursuing imaging for better risk stratification.

The strength of this study includes the relatively large sample size from a single center. Furthermore, we have analyzed BMI both as a continuous variable and as a categorical factor, with the various categories of BMI.

Despite the insights gained, our study has several limitations. First, as a retrospective analysis, it is inherently subject to biases related to data collection and record completeness. Second, the study was conducted at a single tertiary care center, potentially limiting the generalizability of results to broader, more diverse populations. Furthermore, our primary outcome was the association of BMI with tumor grading agreement, and we did not analysis other factors which were deemed beyond the scope of our study question (as mode of biopsy, etc.). Moreover, we did not evaluate the role of other potential confounding factors, such as tumor size, location, or specific histopathological features, which might influence grading accuracy. Lastly, the reliance on preoperative histopathological assessment without adjunctive techniques such as molecular or genetic profiling may have limited the depth of insights into tumor biology.

## Conclusion

Our study found no significant association between BMI and the agreement between preoperative and postoperative tumor grading in EC. Obesity should not be considered as a factor in the accuracy of preoperative endometrial biopsy, and planning for optimal management should take into consideration the preoperative tumor grade, with the knowledge on grading agreement per tumor grade, regardless of patient’s BMI.

## Data Availability

The data that support the findings of this study are not openly available due to reasons of sensitivity and are available from the corresponding author upon reasonable request. Data are located in controlled access data storage at Jthe ewish General Hospital.
